# Metabolomics Reveals Abnormal Citrate Cycle and Phenylalanine Metabolism in Testes from Infertile Hybrid Dzo

**DOI:** 10.3390/ani15203023

**Published:** 2025-10-17

**Authors:** Jiaojiao Ding, Yan Dao, Lingqian Liang, Rui Hong, Huiyou Chen, Yi Yan, Ling Wang, Fuyuan Zuo, Gongwei Zhang

**Affiliations:** 1 College of Animal Science and Technology, Southwest University, Chongqing 402460, China; 13032329910@163.com (J.D.); 13097413946@163.com (Y.D.); llq99lucky@163.com (L.L.); ruihong0101@163.com (R.H.); 17602359737@163.com (H.C.); yy18885601125@163.com (Y.Y.); wling810@163.com (L.W.); zfuyuan@163.com (F.Z.); 2 Beef Cattle Engineering and Technology Research Center of Chongqing, Southwest University, Chongqing 402460, China

**Keywords:** male infertility, α-ketoglutarate, mitochondrial–nuclear incompatibility, TCA cycle, phenylalanine metabolism

## Abstract

**Simple Summary:**

Dzo is a prime example of hybrid male sterility (HMS) in bovines, holding significant theoretical importance in terms of analyzing the mechanisms of interspecific reproductive isolation and speciation. This study revealed that testis-specific abnormalities in the citrate cycle and phenylalanine metabolism are closely associated with dzo male infertility. This research provides comprehensive insights into the citrate cycle as a key pathway linked to dzo sterility, uncovering potential mitochondrial–nuclear incompatibilities that contribute to this HMS phenomenon.

**Abstract:**

This study investigated the metabolomic profiles and molecular basis of hybrid male sterility (HMS) in dzo (the male F1 hybrid offspring of taurine cattle (*Bos taurus*, ♂) × domestic yak (*Bos grunniens*, ♀)). In total, 147 co-different metabolites were identified between liver and testis tissues. Metabolomics analysis linked testis-specific abnormal citrate cycle and phenylalanine metabolism to dzo male infertility. Specifically, α-ketoglutaric acid, L-malic acid, and succinic acid were specific elevated in dzo testes, but not significantly different in liver. The testis-specific metabolite phenyllactate was reduced in dzo. Moreover, genes encoding α-ketoglutarate-dependent oxygenases were dysregulated only in dzo testes, including histone demethylations and RNA m^6^A modifications. Reactive oxygen species and m^6^A content were significantly decreased in dzo testes. Multiomics data showed that testis-specific metabolic abnormalities in dzo were linked to upregulated *IDH3A* and *IDH3G*, and downregulated testis-specific *OGDHL* and *PDHA2*. MiRNA-15b targeting to *IDH3A* was downregulated in dzo testes. The promoter of *PDHA2* was hypermethylated and showed lower chromatin accessibility in dzo testes. Notably, testis-specific *LDHC* downregulation was also associated with lower phenyllactate in dzo testes, which could be an outcome of male infertility. Overall, this study provides comprehensive insights into the citrate cycle as a key pathway associated with dzo sterility, shedding light on the potential mitochondrial–nuclear incompatibility pertinent to addressing this HMS challenge.

## 1. Introduction

In livestock systems, interspecies hybridization or hybridization within species with genetically different traits usually results in heterosis. The hybrid offspring resulting from the cross between taurine cattle (*Bos taurus*, ♂) and domestic yak (*Bos grunniens*, ♀) exhibit significant heterosis in milk yield and meat production. In terms of meat production, these hybrids demonstrate superior carcass yield compared to yak [1], and exhibit an enhanced capacity for protein synthesis than cattle [2]. Furthermore, they possess premium meat characteristics, including higher mineral content, darker coloration, thicker muscle fibers, and distinctive flavor profiles [1,3]. In addition to their meat advantages, these hybrids show a several-fold increase in daily milk yield while maintaining milk quality comparable to that of yaks [4]. However, the male F1 offspring (referred to as ‘dzo’) are sterile, whereas female F1 offspring (‘dzomo’) are fertile. Male sterility in dzo prevents the horizontal fixation of hybrid vigor. Over the past decade, research has focused on investigating dzo sterility, suggesting that this infertility results from meiosis arrest at the pachytene stage [5,6]. Most previous studies focused on RNAs differentially expressed between testis tissue from dzo and cattle or yak, including mRNA [7,8], long noncoding (lnc)RNA [9], miRNA and piwi-interacting (piRNA) [10], and differential abundance proteins [11]. In addition, the levels and distributions of histone methylations differ significantly in meiotic chromosomes of dzo spermatocytes [12]. Differentially methylated genes (e.g., m^6^A) were enriched in pathways related to spermatogenesis in dzo, such as homologous recombination and meiotic spindle organization [13]. Recently, RNA sequencing and assay for transposase-accessible chromatin with high-throughput sequencing (ATAC-seq) analyses suggested the transcription factor *MYBL1* as a candidate master regulator of pachytene spermatocyte genes dysregulated in dzo [7]. Together, these findings highlight the potential roles of transcriptional regulation associated with dzo sterility [7,10].

A previous mRNA sequencing study revealed that most upregulated genes in dzo testes were related to metabolic processes [7]. mRNA-Seq of testis and liver tissues revealed that metabolism-related genes were disordered in dzo testes but not in liver tissues [14]. Metabolites are sensitive indicators of cellular activity reflecting the coordinated and integrated functions of effector molecules that catalyze biochemical reactions. A more recent finding suggest that metabolites have a direct role in transcriptional regulation by influencing chromatin modifications in the nucleus [15]. For example, α-ketoglutarate (also known as 2-oxoglutarate, 2OG) is a citrate cycle (TCA cycle) intermediate and an essential cofactor for many 2OG-dependent oxygenases (2OGX), including Jumonji C (JmjC) Nε-methyl-lysine demethylases (KDM), ten-eleven-translocation (TET) 5-methylcytosine (5mC) hydroxylases, and EGLN prolyl-4-hydroxylases, which regulate hypoxia inducible factor (HIF), and the AlkB homolog family of DNA/RNA demethylases (ALKBH) [16,17]. The biological functions of 2OGXs involve nucleic acid repair, transcriptional/protein biosynthesis regulation, lipid metabolism, hypoxia sensing, and collagen/collagen-related protein biosynthesis [17]. The KDM family of 2OGXs directly remove methyl marks from histones [18,19]. Mammals have nine AlkB homologs (ALKBH1–9), which all catalyze the repair of methylation-induced DNA and RNA damage [20]. Thus, metabolites released from the TCA can regulate histone acetylation [21] and DNA/RNA methylation patterns [22], which act to modulate chromosomal gene expression patterns. However, less is known about the metabolomics changes associated with dzo sterility.

The liver is an essential metabolic organ. Previous work used liver tissue as a proxy for somatic cells and was compared with testis tissue; this approach excluded genes that might be associated with somatic cells in the testes [14], and allowed us to focus on testis-specific disordered metabolites related to dzo infertility. Thus, to better understand the potential association between testis-specific metabolites and dzo sterility, we performed widely targeted ultraperformance liquid chromatography dual mass spectrometry (UPLC-MS/MS)-based metabolite profiling in testes and liver, which represented germ tissues and somatic tissues, respectively. Our results provide insights into the citrate cycle as a key pathway associated with dzo sterility as well as potential mitochondrial–nuclear incompatibility for addressing this hybrid male sterility challenge.

## 2. Materials and Methods

### 2.1. Sample Collection

Testicular tissues from the middle of the testes were obtained from sexually mature taurine cattle (*n* = 8, approximately 36 months), yaks (*n* = 7, approximately 54 months), and dzo (*n* = 9, approximately 54 months) bulls. Since the spermatogenic cycle of adult bulls is constant, these samples can meet the experimental requirements. Liver tissue was collected from cattle (*n* = 6), yak (*n* = 6), and dzo (*n* = 6). In the taurine cattle group, individuals C1, C2, C3, C5, C7, and C8 had both testicular and liver tissues collected; H2 and H3 had only testicular tissues collected. In the yak group, Y1, Y2, Y4, Y5, and Y9 had both testicular and liver tissues collected; Y3 and Y10 had only testicular tissues collected; and Y11 had only liver tissue collected. In the dzo group, CY1, CY2, CY4, CY6, CY7, and HY1 had both testicular and liver tissues collected; while CY3, CY5, and CY8 had only testicular tissues collected. The muscle, spleen, heart, and lung tissues were collected from the cattle, yak, and dzo for the mRNA expression pattern analysis. The individuals from which samples were collected reached market weight in the spring and were subsequently slaughtered at a centralized abattoir. All samples were obtained from a local beef cattle slaughterhouse, with sample collection completed within half an hour after slaughter. Tissue samples were immediately flash-frozen in liquid nitrogen after collection, then stored at −80 °C until further use.

### 2.2. Widely Targeted Metabolomics Analysis

All tissue samples were sent to Metware Biotechnology (Wuhan, China) for widely targeted metabolomics analysis. Sample preparation and extraction, ultra-performance liquid chromatography–electrospray ionization tandem mass spectrometry (UPLC-ESI-QTRAP-MS/MS) were performed according to the manufacturer’s instructions.

#### 2.2.1. Sample Preparation and Extraction

The testis and liver samples were homogenized in a grinder (30 HZ) for 20 s. A 400-μL solution of ice-cold methanol/water (70%, *v*/*v*) containing internal standard (L-2-chlorophenylalanine, [2H5]-Hippuric Acid, [2H5]-Phenoxy acetic Acid) was added to the ground sample, which was then shaken at 1500 rpm for 5 min. The sample was centrifuged at 12,000 rpm for 10 min at 4 °C. Finally, 300 μL of supernatant was collected and placed at −20 °C for 30 min, and then centrifuged at 12,000 rpm for 3 min at 4 °C. Then, 200-μL aliquots of supernatant were transferred for LC-MS analysis.

#### 2.2.2. HPLC Conditions

The sample extracts underwent analysis using an UPLC-ESI-QTRAP-MS/MS system. The samples were injected in the order listed in the collection sheet. A quality control (QC) sample was prepared by mixing the sample extracts, and one QC sample was inserted into every ten test samples to monitor the repeatability of the samples under the same treatment. The specific analytical parameters were configured as follows: UPLC used a Waters Acquity UPLC HSS T3 C18 column (1.8 µm, 2.1 mm × 100 mm), with a column temperature maintained at 40 °C. The flow rate was set at 0.4 mL/min, and the injection volume was 2 μL. The solvent system utilized was a mixture of water (containing 0.04% formic acid) and acetonitrile (containing 0.04% formic acid). The gradient program started with a composition of 95:5 *v*/*v* at 0 min, transitioned to 5:95 *v*/*v* at 11.0 min, reached 5:95 *v*/*v* at 12.0 min, reverted to 95:5 *v*/*v* at 12.1 min, and concluded at 95:5 *v*/*v* at 14.0 min.

#### 2.2.3. ESI-QTRAP-MS/MS

Linear ion trap (LIT) and triple quadrupole (QQQ) scans were acquired using a triple quadrupole-linear ion trap mass spectrometer (Q TRAP), specifically the API 6500 Q TRAP LC-MS/MS system. This system is equipped with an ESI Turbo Ion-Spray interface and operates in both positive and negative ion modes. It is controlled by Analyst 1.6.3 software (AB Sciex, Framingham, MA, USA). The operational parameters of the ESI source were set as follows: source temperature of 500 °C; ion spray voltage (IS) of 5500 V (positive) and −4500 V (negative); ion source gas I, gas II, and curtain gas were adjusted to 55, 60, and 25.0 psi, respectively; the collision gas was maintained at a high level. To ensure optimal performance, instrument tuning and mass calibration were carried out using 10 and 100 μmol/L polypropylene glycol solutions in the QQQ and LIT modes, respectively. For each period, a specific set of multiple reaction monitoring (MRM) transitions was monitored based on the metabolites eluted during that timeframe.

#### 2.2.4. Data Preprocessing and Quality Control

During data preprocessing, missing values are filled with a fixed value of 9, similar to zero-value imputation, to facilitate subsequent statistical analysis. These imputed values are generally not excluded during computation; however, if a substance is missing in all samples within a comparison group (i.e., all values are filled as 9), it cannot be subjected to valid statistical analysis and will therefore be removed from that comparison group. The Total Ion Current (TIC) of QC samples is the sum of the intensities of all ions in the mass spectrum at each time point, with an integration drift error set to 0.1 min.

#### 2.2.5. Qualitative and Quantitative Analysis of Metabolites

For qualitative analysis, primary and secondary MS data were scrutinized using the self-built MWDB database (Metware Biotechnology, Wuhan, China) and publicly available metabolite databases (HMDB, MassBank, METLIN, and MoTo DB, Wuhan, China). Quantitative analysis of metabolites utilized the MRM mode of QQQ MS. The peak area integration was performed on the obtained metabolite MS peaks, and the MS peaks of the metabolites in the different samples were integrated.

### 2.3. Bioinformatics Analysis of Widely Targeted Metabolomics

For widely targeted metabolomics analysis, multivariate statistical analyses of unsupervised principal component analysis (PCA) and hierarchical cluster analysis (HCA) were performed using R software (V4.3.2, www.r-project.org). In addition, Pearson correlation coefficients (PCC) between samples were computed using the cor function in R and depicted solely as heatmaps. Differential metabolites were identified based on the criteria of Variable Importance in Projection (VIP) values (VIP ≥ 1) and absolute Log2FC (|Log2FC| ≥ 1.0). VIP values were extracted from the supervised multiple regression Orthogonal Partial Least Squares Discriminant Analysis (OPLS-DA) results, which included score plots and permutation plots. These plots were generated using the R package MetaboAnalystR. Before OPLS-DA, the data underwent logarithmic transformation (log2) and mean centering. To prevent overfitting, a permutation test comprising 200 permutations was conducted.

Identified metabolites underwent annotation using the Kyoto Encyclopedia of Genes and Genomes (KEGG) Compound database (www.kegg.jp/kegg/compound/, accessed 16 April 2024). Subsequently, the annotated metabolites were mapped to the KEGG Pathway database (www.kegg.jp/kegg/pathway.html, accessed 16 April 2024). The significance of these mappings was assessed through the computation of *p*-values using the hypergeometric test.

### 2.4. Integrative Metabolomics and Transcriptomics Pathway Analysis in Testes

In addition, the two cattle and two dzo testis mRNA-Seq data sets from a previous study [7] were integrated with the metabolomics data sets from the same animals. The clean reads were aligned to the Bos taurus reference genome ARS-UCD1.2 using Hisat2 v.2.0.5 [23]. Read counts were calculated using HTSeq-count. Differentially expressed genes (DEGs) were identified using the DESeq2 R package (v.1.16.0) [24], with the criteria |log2 fold change (FC)| > 1 and *p* < 0.05. Co-KEGG analysis between RNA-seq and metabolomics was performed using ggplot2 in R (V3.5.1). The cornetwork correlation between gene expression and metabolomics was computed using the corr.test function in the R.

### 2.5. m^6^A and Reactive Oxygen Species Content Analysis

An EpiQuik RNA Methylation Quantification Kit (P-9005, Epigentek, Farmingdale, NY, USA) was used to detect mRNA m^6^A levels in cattle, yak, and dzo testes samples based on provided directions. A reactive oxygen species (ROS) ELISA Kit (E004-1-1, NJJCBIO, Nanjing, China) was used to detect ROS content in cattle, yak, and dzo testes samples. The assay kit uses the DCFH-DA (2,7-Dichlorofluorescin Diacetate) probe to detect intracellular reactive oxygen species (ROS) levels, with results expressed in arbitrary units (AU). All samples were maintained at the same cell density during the experiment, and the animal samples used were the same batch as those used for metabolomics analysis.

### 2.6. LDHC mRNA Expression and Association with Phenyl Lactate Content

RNA was extracted from the ground tissue using the Trizol method, and cDNA was synthesized via a PrimeScript™ RT reagent Kit (RR037A, Takara, Kyoto, Japan). Target gene expression in different tissues was determined in testes, muscle, liver, spleen, heart, ovary, kidney, and lung tissues using reverse transcription PCR (RT-PCR) as described in a previous study [5]. The mRNA expression of the target gene *LDHC* was determined by RT-quantitative PCR (qPCR) in cattle, yak, and dzo testes as described in previous study [5]. Primers for all these genes were designed using Primer3 software and synthetized by Genewiz (Suzhou, China). Relative mRNA expression was analyzed with the relative quantification software of the system based on the 2^delta-delta-Ct^ method (Bio-Rad, Hercules, CA, USA). Graphs for mRNA expression differences were produced with GraphPad Prism 9.3. The association between *LDHC* mRNA expression and phenyl lactate content was determined using the power function in Excel.

### 2.7. miRNA-Seq and Target miRNA Prediction for IDH Genes

miRNA expression patterns in dzo, cattle, and yak testes were determined according to previous small RNA-seq data [10]. To estimate miRNA levels in each sample, mapping read data were quantified as transcripts per million reads [TPM = (readCount × 1,000,000)/total readCount] to calculate and normalize expression [25]. Differential expression analysis was performed using DESeq2 to compare the two groups [24]. miRNAs with adjusted *p* < 0.05 were classified as DE miRNAs. Venn diagrams of DE miRNA were produced using the Bioinformatics & Evolutionary Genomics website (https://bioinformatics.psb.ugent.be/webtools/Venn/, accessed 1 May 2024)). The TargetScan algorithm was used to predict the target miRNA of the bovine IDH and LDH gene 3′-untranslated regions (UTRs) (www.targetscan.org/vert_72/, accessed 4 May 2024).

### 2.8. DNA Methylation Levels and Chromatin Accessibility in PDHA2 Promoters

The DNA methylation levels and chromatin accessibility status of the metabolism-associated genes in dzo and cattle testes were determined according to previous whole-genome bisulfite-seq data [10] and ATAC-seq data [7]. Each target gene was visualized using the Integrative Genomics Viewer (V2.4.10, www.broadinstitute.org/igv, accessed 12 May 2024).

### 2.9. Statistical Analysis

RT-qPCR data were expressed as mean ± SD. For all statistical analyses, *p* < 0.05 was considered significant. One-way ANOVA and Tukey’s multiple comparisons tests were conducted for all statistical significance analyses using GraphPad Prism 8.0 software.

## 3. Results

### 3.1. Global View of Metabolites Data

**Figure 1:**
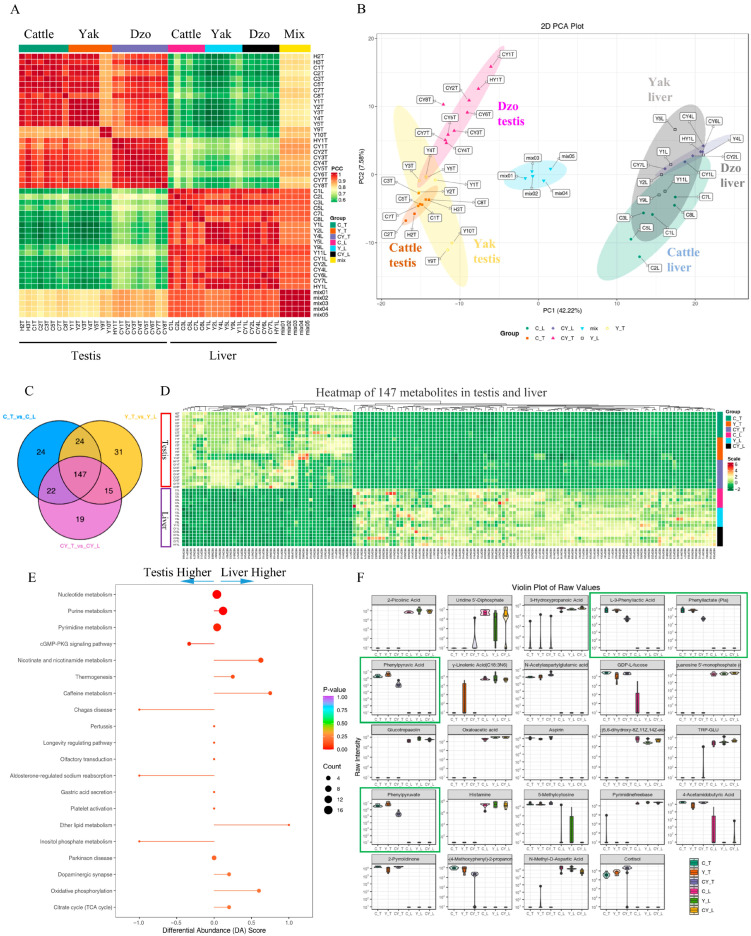
Global and differential metabolite abundance between liver and testis tissues in cattle, yak, and dzo. (**A**) Pearson correlation coefficient within the mixed samples and each group. (**B**) Principal component analysis (PCA) reveals a clear separation between testis and liver groups. (**C**) Venn plot of different metabolites between liver and testis tissues across all three groups (cattle, yak, and dzo). (**D**) Heatmap of 147 different metabolite profiles between testis and liver tissues in all three groups. (**E**) Pathway enrichment of the differentially abundant metabolites in (**D**). (**F**) Tissue-specific metabolites identified in testis and liver.

In total, 481 metabolites were identified in testes and liver of cattle, yak, and dzo (Table S1). These 481 metabolites were assigned into 33 categories, including 97 amino acid metabolomics, 87 organic acids and their derivatives, 56 nucleotide metabolomics, 34 benzenes and substituted derivatives, 33 carbohydrate metabolomics, 28 lipids fatty acids, 18 lipids others phospholipid, 15 co-others enzyme factors and vitamins, 13 oxidized lipids, ten fatty acyls, nine alcohols, eight lipids, seven indoles and their derivatives, seven organic acids and their derivatives, and others (Figure S1A). These metabolites comprised 87% of the 481 total metabolites. Pearson correlation coefficients within the quality control samples (named as mix) were >0.995. Each individual sample within each group had high Pearson correlation coefficients (R^2^ ˃ 0.93), except two yak (Y9T and Y10T) testes tissue samples (R^2^ = 0.85 to 0.87, Figure 1A). Nevertheless, the correlation coefficient between Y9T and Y10T was still above 0.99, indicating a high degree of similarity between the two. The underlying causes for the differences observed in Y9T and Y10T compared to the other samples may stem from sampling errors, sequencing biases, or biological individual variation. Although these two yak testis samples differ from the others, the high reproducibility within the group ensures that the reliability of the intra-group results is not affected. Meanwhile, this variation also provides useful information for further exploring individual differences and optimizing experimental procedures. In summary, these data indicate that there was a good repeatability of each group.

### 3.2. Differentially Abundant Metabolites Between Liver and Testis Tissues in Bovine

To reveal differences in the metabolite profiles of liver and testis tissues in bovine, PCA and OPLS-DA were used. PCA showed that the cluster of the mix group was equally separate from the testis and liver groups (Figure 1B). These 42 samples clearly clustered in the PC1 space, accounting for 42.22% of the variance (Figure 1B), which indicated significant differences between testis and liver tissues. OPLS-DA can maximize the discrimination between groups and is helpful for identifying different metabolites. OPLS-DA identified that 59.1% of the variance was associated with the predictive component in cattle, with R2X = 0.662, R2Y = 0.999, Q2Y = 0.987 (Figure S1B), whereas 52.8% of the variance was associated with the predictive component in yak, with R2X = 0.665, R2Y = 0.998, Q2Y = 0.991 (Figure S1C). These results showed that the OPLS-DA model was stable and efficient.

The VIP from the OPLS-DA analysis model can preliminarily screen out metabolites that differ among different varieties or tissues. In this study, VIP ≥ 1 combined with a fold change ≥2 or ≤0.5 was used to further screen out differential metabolites. This showed that 217, 217, and 203 metabolites were significantly different between liver and testes from cattle, yak, and dzo, respectively (Figure S1D). Among these, 147 metabolites co-differed between liver and testes in these three groups (Figure 1C,D). These metabolites were enriched in purine metabolism, cGMP-PKG signaling pathway, nicotinate and nicotinamide metabolism, oxidative phosphorylation, pyrimidine metabolism, and citrate cycle (TCA cycle), among others (Figure 1E). Interestingly, 27 metabolites were tissue specific (Figure 1F, Table S1). Deoxyguanosine, L-3-phenyl lactic acid, phenyl lactate (Pla), phenylpyruvic acid, *d*-acetylaspartylglutamic acid, GDP-L-fucose, aspirin, phenylpyruvate, 5-methylcytosine, 4-acetamidobutyric acid, 2-pyrrolidinone, 1-(4-methoxyphenyl)-2-propanone, and cortisol occurred only in testes. By contrast, 2-picolinic acid, uridine 5′-diphosphate, 3-hydroxypropanoic acid, γ-linolenic acid (C18:3N6), deoxyguanosine 5′-monophosphate (dGMP), glucotropaeolin, oxaloacetic acid, (±)5,6-dihydroxy-8Z,11Z,14Z-eicosatrienoic acid, TRP-GLU, histamine, *N*-isovaleroylglycine, *N*-propionylglycine, pyrimidinefreebase, and *N*-methyl-D-aspartic acid only occurred in liver.

### 3.3. Differentially Abundant Metabolites Between Fertile and Infertile Testis Tissues

**Figure 2:**
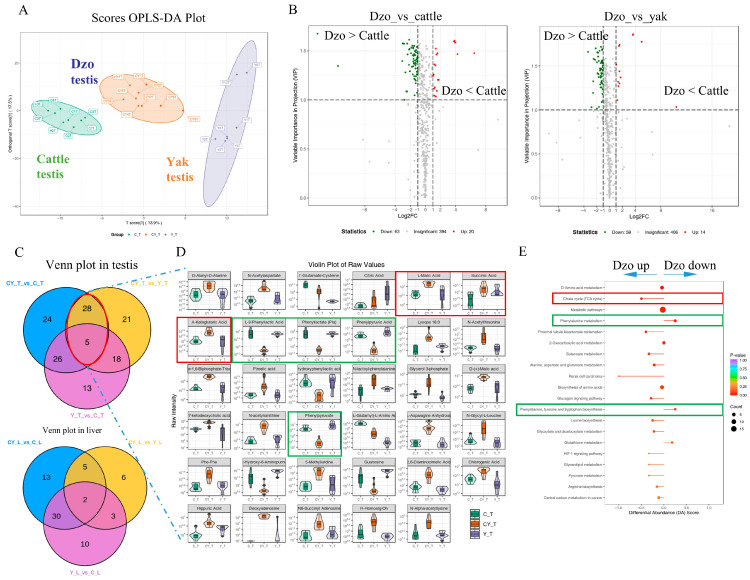
Differential metabolite abundance between fertile and infertile testis tissues. (**A**) Orthogonal Partial Least Squares–Discriminant Analysis (OPLS-DA) analysis between testes from fertile cattle and yak compared with infertile dzo. (**B**) Volcano plot of significantly different metabolites between dzo and cattle, and yak testis, respectively. (**C**) Venn plot of differentially expressed metabolites between dzo and cattle or yak in testes (top) and liver (bottom). (**D**) Violin plot of the 33 differentially expressed metabolites between dzo and cattle, and yak testis. (**E**) KEGG pathways of the 33 differentially expressed metabolites from (**D**).

OPLS-DA was used to search for differences in the metabolite profiles between testis tissues from fertile and infertile phenotypes. Cattle and yak were clearly spaced separately from dzo with an orthogonal T score of 13.9% (Figure 2A). Here, 83 and 72 metabolites were significantly different between dzo and cattle and between dzo and yak testes, respectively (Figure 2B). Among these, 33 metabolites co-differed between dzo and cattle and yak testes (Figure 2C, top), as revealed by a violin plot (Figure 2D). However, only seven metabolites co-differed between dzo and cattle and yak liver (Figure 2C, bottom). Interestingly, the production levels of L-malic acid (MEDN200), succinic acid (MEDN201), and α-ketoglutaric acid (MEDN202) were elevated in dzo testes compared with cattle and yak (Figure 2D, red box). However, these three metabolites of citrate cycle did not differ significantly between dzo and cattle and yak in liver tissue. The testis-specific metabolites, L-3-phenyllactic acid (MEDN324), Pla (MEDN338), phenylpyruvic acid (MEDN339), and phenylpyruvate (MEDN862) were reduced in dzo testes (Figure 2D, green box). These metabolites were enriched in the citrate cycle (TCA cycle), phenylalanine metabolism, HIF-1 signaling pathway, glutathione metabolism, and D-alanine metabolism KEGG pathways (Figure 2E).

### 3.4. α-Ketoglutarate-Dependent Oxygenase mRNA Expression Upregulated in Dzo Testes

**Figure 3:**
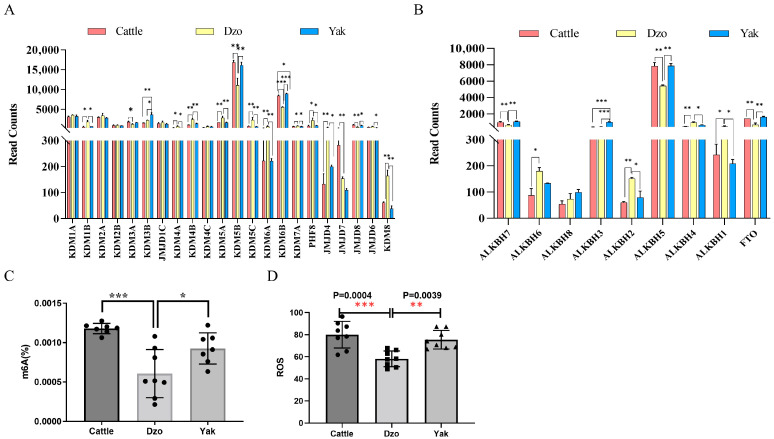
α-Ketoglutarate-dependent oxygenase mRNA expression and m^6^A content. Expression of KDMs (**A**) and ALKBHs (**B**) in dzo compared with cattle or yak. Data from previous RNA-seq study [7]. (**C**,**D**) ROS and m^6^A content in dzo compared with cattle or yak. * *p* < 0.05; ** *p* < 0.01; *** *p* < 0.001.

Metabolites from the TCA cycle can function as epigenetic cofactors to initiate transcriptional reprogramming [26], while ROS from mitochondrial respiration act as signaling molecules or protein oxidation modifiers to regulate transcription and protein activity [27]. Testis and liver RNA-seq data showed that most KDM and ALKBH genes were significantly upregulated in dzo testes compared with cattle or yak (Figure 3A,B), whereas there were no significant differences in liver. Members of the KDM family, including *KDM1B*, *KDM4A*, *KDM4B*, *KDM5A*, *KDM5C*, *KDM6A*, and *PHF8*, were upregulated, whereas *KDM5B* and *JMJD8* were downregulated, in dzo testes (Figure 3A). Among the ALKBH family, *ALKBH5*, *ALKBH7*, and *ALKBH9 (FTO)* were downregulated in dzo testes (Figure 3B). m^6^A and ROS contents were significantly decreased in dzo testes compared with cattle (Figure 3C,D). Previous work revealed that the level and distribution of histone methylations were strikingly different in meiotic chromosomes of dzo spermatocytes [12]. m^6^A modification level was different between dzo and yak testes [13]. Thus, an abnormal TCA cycle might function as an epigenetic cofactor leading to meiotic arrest in dzo.

### 3.5. Transcriptomic and Metabolomics Integrative Analysis Reveals IDH and PDHA2 Genes Were Associated with Citrate Cycle Disorder in Dzo Testes

**Figure 4:**
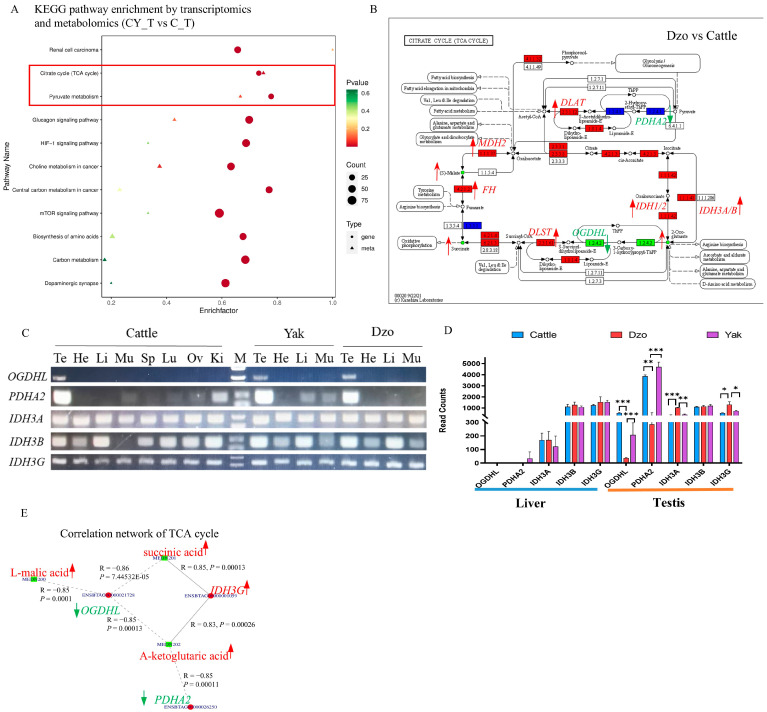
Integrative analysis of transcriptomics and metabolomics indicates association of citrate cycle with dzo sterility. (**A**) Differentially expressed genes and metabolites are co-enriched in KEGG pathways, including the citrate cycle (TCA cycle), pyruvate metabolism, renal cell carcinoma, HIF-1 signaling pathway, carbon metabolism, and biosynthesis of amino acids, among others (adjusted *p*-value of gene < 0.05). (**B**) Within the citrate cycle (TCA cycle) pathway, the abundance of L-malic acid, succinic acid, and α-ketoglutaric acid positively correlates with gene expression. ↑ denotes upregulated genes; ↓ indicates downregulated expression. (**C**) RT-PCR reveals widespread expression of IDH genes (*IDH3A*, *IDH3B*, and *IDH3C*) in various tissues, whereas *OGDHL* and *PDHA2* are specifically expressed in the testis. (**D**) RNA-seq data demonstrate upregulated expression of *IDH3A* and *IDH3G* in dzo testis, with no significant differences in the liver. Conversely, *OGDHL* and *PDHA2* show testis-specific downregulation in dzo testis (* *p* < 0.05; ** *p* < 0.01; *** *p* < 0.001). (**E**) Correlation network of transcriptomics and metabolomics reveals significant positive or negative correlations between *IDH3G*, *OGDHL*, and *PDHA2* mRNA expression and L-malic acid (MEDN200), succinic acid (MEDN201), and α-ketoglutaric acid (MEDN202).

An integrative analysis of both the metabolome and transcriptome was conducted to understand the core genes that affect the production of differential metabolites between dzo and cattle testes. We found that DEGs and the metabolome are co-enriched in the KEGG pathways of the citrate cycle (TCA cycle), pyruvate metabolism, renal cell carcinoma, HIF-1 signaling pathway, carbon metabolism, and biosynthesis of amino acids (Figure 4A, adjusted *p*-value of gene < 0.05). Among these enriched pathways, L-malic acid (MEDN200), succinic acid (MEDN201), and α-ketoglutaric acid (MEDN202) are involved in the TCA cycle, pyruvate metabolism, carbon metabolism, biosynthesis of amino acids, and HIF-1 signaling pathway. In the TCA cycle pathway, the abundance of L-malic acid, succinic acid, and α-ketoglutaric acid was positively correlated with gene expression (Figure 4B). Three forms of isocitrate dehydrogenase (IDHA, IDHB, and IDHG) catalyze the oxidative decarboxylation of isocitrate to α-ketoglutarate. Oxoglutarate dehydrogenase (OGDH) catalyzes the overall conversion of 2OG to succinyl-CoA. Pyruvate dehydrogenase E1 alpha (PDHA) is an essential and rate-limiting enzyme in aerobic glucose metabolism and catalyzes the conversion of pyruvate to acetyl-CoA, connecting glycolysis to the oxidative citrate cycle. RT-PCR revealed that IDH genes were widely expressed in a variety of tissues, whereas *OGDHL* and *PDHA2* were only found in testes as part of the testis-specific pyruvate dehydrogenase complex (Figure 4C). RNA-seq data showed upregulated expression of *IDH3A* and *IDH3G* in dzo testes but no significant difference in liver. However, *OGDHL* and *PDHA2* showed testis-specific downregulated expression in dzo testes (Figure 4D). Meanwhile, correlation analysis of transcriptomics and metabolomics showed that *IDH3G*, *OGDHL*, and *PDHA2* mRNA expression was significantly positively or negatively correlated with L-malic acid (MEDN200), succinic acid (MEDN201), and α-ketoglutaric acid (MEDN202) (Figure 4E). These results revealed that abnormal gene expression and content of citrate cycle are associated with dzo sterility.

### 3.6. Testis-Specific LDHC mRNA Downregulation Associated with Lower Phenylalanine Metabolism in Dzo Testes

**Figure 5:**
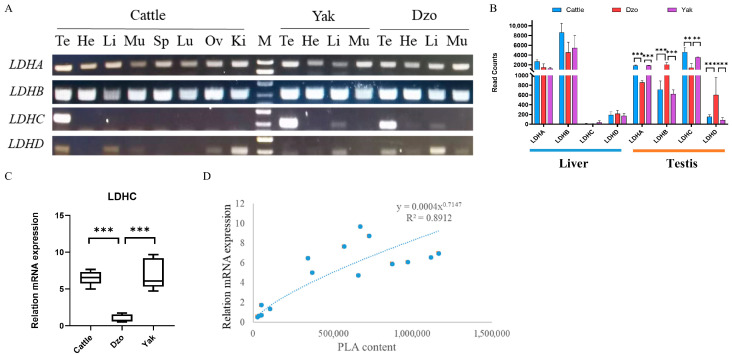
Testis-specific *LDHC* mRNA downregulation associated with reduced phenylalanine metabolism in dzo testes. (**A**) Tissue expression patterns of target genes using RT-PCR. (**B**) RNA-seq data of LDH genes in liver and testes from a previous study [7]. (**C**) Fold changes in *LDHC* mRNA expression in testis using RT-qPCR. (**D**) Power function plot of association between *LDHC* mRNA expression and phenyllactate content. ** *p* < 0.01; *** *p* < 0.001.

**Figure 6:**
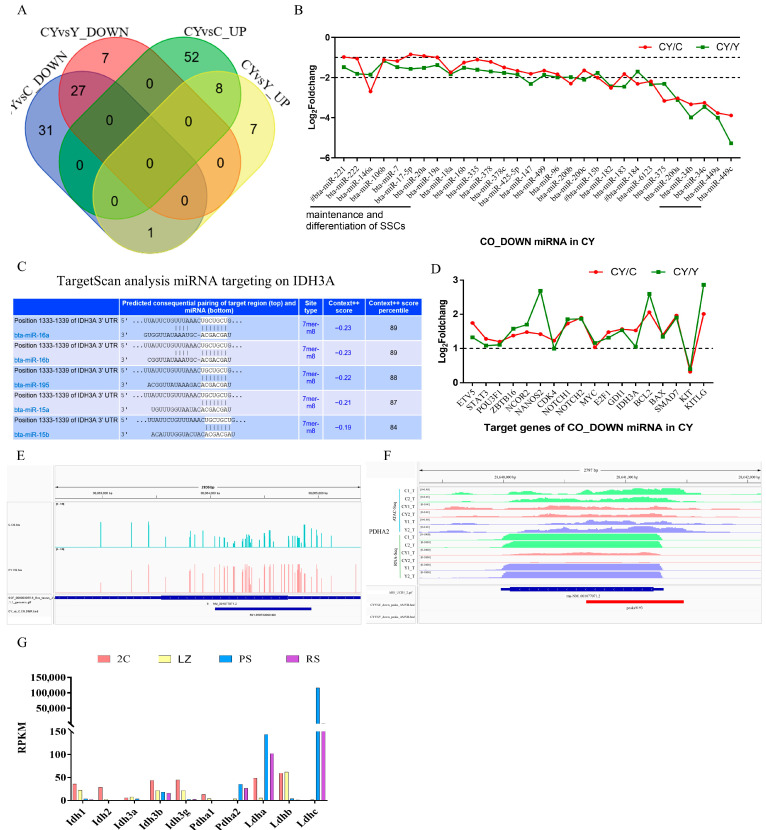
Epigenetic factors regulate *IDH3A* and *PDHA2* expression. (**A**) Venn plot of miRNAs differentially expressed between dzo and cattle or yak in testes. (**B**) Fold-change in decreased expression of miRNA between dzo and cattle or yak in testes. (**C**) Prediction of binding sites between miRNA and the *IDH3A* 3′UTR. (**D**) Fold-change in the decreased expression of target gene miRNA. (**E**) DNA methylation levels of *PDHA2* in dzo and cattle testes based on previous whole-genome bisulfite sequencing data [10]. (**F**) Track views of ATAC-seq and RNA sequencing of *PDHA2* in cattle, yak, and dzo testes based on previous ATAC-seq data [7]. (**G**) Expression patterns of *IDH* and *LDH* genes in purified mouse spermatogenic cell populations. Abbreviations: 2C, most contained somatic cells; LZ, leptotene and zygotene spermatocytes; PS, pachytene spermatocytes; RS, round spermatids. Data from da [33].

L-3-phenyllactic acid (MEDN324), Pla (MEDN338), phenylpyruvic acid (MEDN339), and phenylpyruvate (PPA, MEDN862) were testis-specific metabolites (Figure 1F, green box) and were significantly reduced in dzo testes (Figure 2D, green box). These metabolites were enriched in the phenylalanine metabolism KEGG pathway. PLA is synthesized via the amino acid metabolic pathway, in which phenylalanine is catabolized to phenylpyruvate (PPA) by transaminase and then reduced to Pla by lactate dehydrogenase (LDH) [28]. LDH has a crucial role in the final production of Pla. There are four isozymes of LDH in cattle: LDHA, LDHB, LDHC, and LDHD. LDHA and LDHB are involved in anaerobic metabolism for pyruvate reduction and for aerobic oxidation of lactate, respectively; LDHC is a testis-specific isozyme expressed in testes and spermatozoa [29]. The tissue expression patterns showed that *LDHA* and *LDHB* were widely expressed in all tested tissues, whereas *LDHC* showed testis-specific expression (Figure 5A). RNA-seq data and RT-qPCR showed that *LDHA* and *LDHC* were downregulated in dzo testes, but not significantly different in liver (Figure 5B,C). Power function association analysis showed a significant correlation between *LDHC* mRNA expression and Pla content (Figure 5D). These results suggest that testis-specific downregulated *LDHC* is associated with lower testis-specific Pla content. LDHC is primarily expressed in pachytene spermatocytes (PS) and round spermatids (RS) of mouse spermatogenic cells (Figure 6G). In dzo, due to meiotic arrest during spermatogenesis, there are almost no pachytene spermatocytes or subsequent stage cells present in the seminiferous tubules, accompanied by widespread germ cell apoptosis; therefore, the downregulation of LDHC expression is a key manifestation of this abnormal process.

### 3.7. miRNA and DNA Methylation Associated with Regulating Citrate Cycle Disordered in Dzo Testes

An integrative analysis of miRNA, DNA methylation, and ATAC data from previous studies [7,10] was conducted to understand the regulatory factors that affect core gene expression and production of α-ketoglutaric acid and Pla metabolites in dzo versus cattle testes. Based on the miRNA data, 27 and eight miRNAs showed decreased and increased expressed in dzo testes (Figure 6A), respectively. Among the 27 miRNAs showing decreased expression, the degree of the downregulation of male germ cell stage-specific miRNA expression during spermatogenesis gradually increased (Figure 6B). The TargetScan algorithm suggested *IDH3A* as a target gene of miR-15a/b and miR-16a/b (Figure 6C); *ODGHL* for miR-9-5p and miR-200b/c; *PDHA2* for miR-204; *LDHA* for miR-34/449; and *LDHC* for miR-200b/c (). miR-15b, miR-16b, miR-200a/b/c, miR-34b/c, miR-449a/c, and miR-9-5p showed significantly decreased expression in dzo testes (Figure 3B). Previous work revealed that miR-15b targets the genes encoding GDP dissociation inhibitor 1 (*GDI1*) and isocitrate dehydrogenase 3 [*NAD* (+)] alpha (*IDH3A*), and regulates TCA cycle-mediated energy metabolism [30]. miR-221/222 promote the stemness characteristics of spermatogonial stem cells and both miR-20 and miR-106a directly target STAT3, which has crucial roles in the maintenance and renewal of SSCs; members of the miR-34 family and miR-449 regulate *NOTCH1* and *BCL2* (reviewed by [31]). Indeed, the expression of target genes of these miRNAs were significantly upregulated in dzo testes (Figure 6D). These results highlight the importance of male germ cell stage-specific miRNAs in regulation of the TCA cycle and somatic cells in spermatogenesis.

Basing on previous WGBS and ATAC-seq data [7,10], the promoter of *PDHA2* was hypermethylated and chromatin accessibility was decreased in dzo testes compared with cattle (Figure 6E,F). Interestingly, male meiosis-specific master transcription factor MYBL1 binding motifs were identified in the promoter of *PDHA2* (Figure 6F). In addition, DNA methylation and chromatin accessibility were not significantly different in IDH3A and LDHC. Previous work revealed that MYBL1 directs germ cell-specific activation via the cAMP-responsive element sites of *PDHA2* and *LDHC*, which are activated specifically in primary spermatocytes [32]. These results suggest that DNA methylation and transcription factor MYBL1 are associated with regulating *PDHA2* and *LDHC* expression. In future studies, the potential involvement of the transcription factor MYBL1 in regulating PDHA2 and LDHC expression can be validated by interfering with or overexpressing MYBL1 and subsequently observing changes in PDHA2 and LDHC expression. Additionally, the impact of altered PDHA2 promoter methylation status on MYBL1 binding and gene expression can be assessed.

It was not possible to obtain sufficiently purified spermatogenic cell populations to investigate the stage-specific expression of these TCA and LDH genes in domestic cattle. Data from mice [33] showed that IDH genes and *LDHB* are turned on in most somatic cells (2C), and maintain a higher level in leptotene or zygotene spermatocytes (LZ), whereas expression of *PDHA2* and *LDHC* peaked in pachytene spermatocytes (PS) (Figure 6G). Taken together, downregulation of miRNA might be associated with upregulation of the TCA cycle and somatic cells in dzo testes, whereas hypermethylated DNA and decreased chromatin accessibility in early meiosis genes downregulate pachytene spermatocytes gene expression, with a decrease in Pla metabolites being involved in the arrest of meiosis at the pachytene stage.

## 4. Discussion

The heterosis of meat and milk production in dzo is obvious, whereas male infertility remains unexplained. Most DEGs associated with somatic cell (such as Sertoli cells) functions have been found in dzo testes [7,34]. Previous work found no significant difference in the expression of metabolic process genes in dzo liver compared with cattle or yak (−1 ≤ log2FC ≤ 1). However, the expression of these genes was disordered in dzo testis tissues (−3 ≤ log2FC ≤ 3). Testis-specific metabolic gene expression was also reported to be disordered in dzo [14]. Therefore, this study used the liver as a comparison somatic tissue and as a means of identifying the testes-specific differential abundance of metabolites associated with dzo infertility.

Due to meiotic arrest, spermatogenic cells in dzo undergo apoptosis, resulting in seminiferous tubules that contain only residual spermatogonia and a small number of spermatocytes, with an absence of spermatids; in contrast, the seminiferous tubules of yak contain all types of germ cells [35]. Comparison of the metabolic profiles of the liver and testes in three types of bovines revealed notable metabolic differences. In the dzo liver, there were only seven common differentially abundant metabolites compared with cattle and yaks. However, in testes tissue, dzo exhibited a more pronounced difference, featuring 33 distinct differentially abundant metabolites. The differences in metabolites between testicular tissues are closely related to the differences in their cellular composition. Compared with cattle and yaks, the levels of L-malic acid, succinic acid, and α-ketoglutaric acid were significantly elevated in the testes of dzo, signifying a disturbance in the TCA cycle. Notably, these three TCA metabolites did not display significant differences in the liver tissue between dzo and cattle or yaks. Meanwhile, ROS was significantly decreased in the testes of dzo (Figure 6D). Mitochondria are known as the ‘powerhouse’ of the cell, and produce ATP through the TCA cycle and oxidative phosphorylation (OXPHOS), which has a role in spermatogenesis [27]. ROS that are generated during OXPHOS can induce protein oxidative modifications or serve as secondary messengers to trigger signaling cascades [36]. These results revealed that mitochondria metabolism was specifically disrupted in the dzo testicular tissue.

High compatibility between mitochondrial and nuclear genomes is important to maintain structural and biochemical properties required for OXPHOS function [37], which undergo adaptive coevolution to maintain energy metabolism [38]. Thus, interspecies incompatibility of mitochondrial and nuclear genomes could represent disruptions to an interspecies-generated OXPHOS system [39]. Dzo derived from cross breeding *B. taurus* (♂) and *B. indicus* (♀), result in hybrid cattle nuclear and yak mitochondrial DNA. A previous study using transmitochondrial cybrids (cytoplasmic hybrids) found that the yak cybrid (cattle nuclear + yak mitochondrial) had a lower oxygen consumption rate and extracellular acidification rate, lower ATP turnover and glycolytic reserve, lower mitochondrial DNA copy numbers, and lower mRNA expression of mitochondrial biogenesis-related genes compared with the cattle cybrid (cattle nuclear + cattle mitochondrial) [40]. Thus, these result reveal perturbations in mitochondrial function and energy metabolism in the yak cybrid [40]. Therefore, future work should investigate any interspecies incompatibility of the mitochondrial and nuclear genomes in dzo.

Metabolites that are produced in the TCA cycle, such as α-ketoglutarate, serve as cofactors for epigenetic regulators that modulate chromatin modifications and DNA/RNA methylation [17,21,22]. The current study found upregulation of several genes within the KDM family and downregulation of genes in the ALKBH family in dzo testes. KDMs influence histone demethylation [18,19]. These results suggest that an abnormal citrate cycle is associated with histone and m^6^A modification. Previous work showed that the level of H3K36me3 was significantly decreased in dzo testes compared with yaks, but not of H3K27me3 [41]. Depletion of H3K4me3 and significant enrichment of H3K27me3 and H4K20me3 were also observed in Sertoli cells from dzo. In addition, strikingly different levels and localizations of H3K4me3, H3K9me1, H3K9me3, and H4K20me3 were noted in meiotic chromosomes of dzo spermatocytes [12]. Previous research showed that m^6^A erasing genes (*ALKBH5* and *FTO*) were specifically downregulated in infertile Dzo testes, but not in liver and muscle tissues [42]. In the current study, the m^6^A content was significantly decreased in dzo testes comparing with cattle testes. Differentially methylated m^6^A genes are associated with spermatogenesis, homologous recombination, apoptosis, and steroid hormone biosynthesis [13]. Thus, the current findings suggest that the atypical α-ketoglutarate content mediates the abnormal expression of 2OGX genes during dzo spermatogenesis. The mechanism might involve an impact on histone methylation and m^6^A modification of RNA, ultimately leading to male infertility in Dzo. Utilizing the liver as a control group, the current study identified dzo testis-specific differential metabolites and, for the first time, established the relationship between the intermediate metabolite α-ketoglutarate in the TCA cycle and the expression of testicular meiotic genes. This provides crucial molecular evidence for unraveling the molecular mechanisms of male infertility in dzo.

To gain deeper insights into the alterations in metabolites within the TCA cycle, we performed an exhaustive analysis that integrated transcriptomic and epigenetic data. IDH facilitates the oxidative decarboxylation of isocitrate, producing α-ketoglutarate. Subsequently, *OGDH* oversees the comprehensive conversion of α-ketoglutarate to succinyl-CoA. Our RNA-seq data revealed upregulation of the expression of *IDH3A* and *IDH3G* in dzo testes, whereas no significant differences were observed in the liver. Conversely, the testis-specific expression of *OGDHL* in dzo testes showed a significant decrease. These findings offer an explanation for the elevated levels of α-ketoglutarate in Dzo testes, because it cannot be converted to succinyl-CoA to propel the TCA cycle. Concurrently, the diminished expression of *OGDHL* and *PDHA2*, constituents of the testis-specific pyruvate dehydrogenase complex, would result in decreased levels of acetyl-CoA from pyruvate and succinyl-CoA from α-ketoglutarate. This leads to a reduction in acetyl-CoA entering the TCA cycle, along with decreased conversion of the upstream substrate α-ketoglutarate to downstream succinate and L-malic acid. These alterations are consistent with the observations in the metabolomics data from dzo testes. Consequently, this could bring about a decrease in NADH generated through the TCA cycle, ultimately leading to reduced ATP production. Insufficient ATP levels are known to be crucial for spermatogenesis, contributing to the infertility observed in male Dzo.

Interestingly, a range of male germ cell stage-specific miRNAs were gradually downregulated during spermatogenesis (Figure 6B), and the target genes of these miRNAs were upregulated in dzo testes (Figure 6D). Previous RNA-seq data showed that metabolic process, apoptosis, and spermatogonia proliferation genes were upregulated in dzo [7]. These results suggest downregulation of male germ cell stage-specific miRNA to be important in the TCA cycle and somatic cell development in dzo.

Previous work showed that regulation of *PDHA2* expression is mediated by proximal promoter sequences and CpG methylation [43]. The promoter of the pachytene gene *PDHA2* was hypermethylated and chromatin accessibility was decreased in dzo testes compared with cattle (Figure 6E,F). Earlier work revealed that the promoter of piRNA metabolism and meiosis I genes were hypermethylated in dzo testes [10]. Prepachytene piRNAs direct DNA and histone methylation at transposon sequences [44,45], which are increased in dzo testes [10]. In addition, specific to the testes, there is a notable downregulation of metabolites, including L-3-phenyllactic acid, Pla, phenylpyruvic acid, and phenylpyruvate, in Dzo. The breakdown of phenylalanine involves transaminase catalyzing its decomposition into phenylpyruvic acid (PPA), subsequently reduced to Pla by LDH [46]. LDHC, a crucial enzyme in Pla production, exhibits specific expression in the testes and is significantly downregulated in dzo, while showing no significant difference in the liver. This suggests the involvement of LDHC in Pla synthesis. Prior research indicated that MYBL1 does not directly bind to the *LDHC* and *PDHA2* gene promoter sequences [47]. Instead, MYBL1 directs germ cell-specific activation via the CRE site of certain genes activated specifically in the primary spermatocyte [32]. Earlier work showed that testis-specific MYBL1, possibly a key transcription factor contributing to extensive gene expression disruption in dzo spermatocytes during the pachytene stage, experiences downregulation in the testes of dzo [7]. *LDHC* and *PDHA2* showed higher expression in the pachytene stage (Figure 6G), implying that the decreased expression of *LDHC* and *PDHA2* is the result of a few specific spermatogenic cells expressing such genes in dzo testes. Consequently, the significant downregulation of testis-specific metabolites, including L-3-phenyllactic acid, Pla, phenylpyruvic acid, and phenylpyruvate, in the testes of Dzo might also be the outcome of male infertility.

## 5. Conclusions

**Figure 7:**
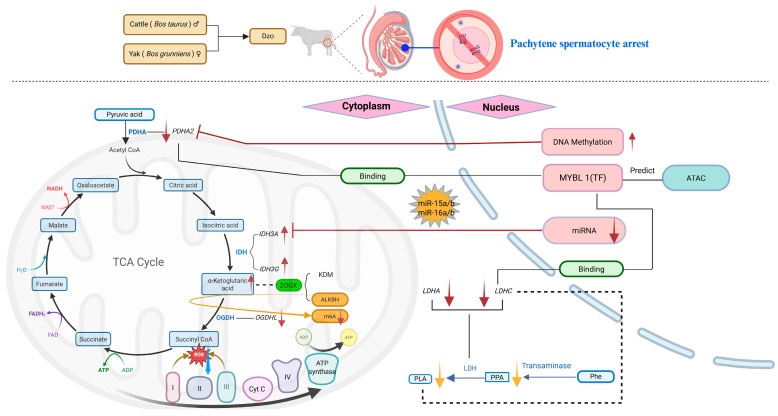
The potential mitochondrial-nuclear incompatibility underlying hybrid male sterility in dzo. Metabolism-related elements are highlighted with blue boxes and blue text, and the involved genes are indicated in italics. (BioRender, https://BioRender.com).

Overall, based on the metabolomics and multiomics data, we hypothesis that male germ cell stage-specific miRNA regulates TCA cycle and mitosis; as an epigenetic cofactor, α-ketoglutaric acid affects histones and m^6^A modification; and DNA hypermethylation, transcription factor MYBL1, and chromatin accessibility affect meiosis arrest in pachytene spermatocytes (Figure 7). However, further research is needed to determine the causal factors leading to the dysregulation of male mitotic-stage-specific miRNA expression and meiotic DNA hypermethylation in dzo testes.

## Data Availability

None of the data were deposited in an official repository. The data that support the experiment findings are available from the authors upon request.

## Supplementary Materials

The following supporting information can be downloaded at: https://www.mdpi.com/article/10.3390/ani15203023/s1, Figure S1: The distribution of identified 481 metabolites (A); Scores OPLS-DA Plot (B,C); Differential Metabolite Screening (D); Table S1: Table of 27 tissue-specific metabolites in testis and liver.

## Abbreviations

The following abbreviations are used in this manuscript:

TCA	citrate cycle
2OGX	2OG-dependent oxygenases
KDM	lysine demethylases
HIF	hypoxia inducible factor
ALKBH	AlkB homolog family
DEGs	differentially expressed genes
Pla	phenyl lactate
ROS	reactive oxygen species
OGDH	oxoglutarate dehydrogenase
2OG	α-ketoglutarate or 2-oxoglutarate
PDHA	pyruvate dehydrogenase E1 alpha
LDH	lactate dehydrogenase
IDH3A	isocitrate dehydrogenase 3 [NAD (+)] alpha
LZ	leptotene or zygotene spermatocytes
PS	pachytene spermatocytes
OXPHOS	oxidative phosphorylation
piRNA	piwi-interacting RNA
PCA	principal component analysis
OPLS-DA	orthogonal partial least squares discriminant analysis
VIP	variable importance in projection
KEGG	Kyoto Encyclopedia of Genes and Genomes
