# Supplementary material for: Metabolomics Reveals Abnormal Citrate Cycle and Phenylalanine Metabolism in Testes from Infertile Hybrid Dzo
Source: Animals (Basel). 2025 Oct 17;15(20):3023. doi: 10.3390/ani15203023 (PMC12562072; doi:10.3390/ani15203023)
Supplement: Supplementary file 1 [file animals-15-03023-s001.zip › animals-3893473-supplementary.pdf]

Metabolomics Reveals Abnormal Citrate Cycle and Phenylalanine Metabolism in Testes from Infertile Hybrid Dzo

Jiaojiao Ding [1], Yan Dao [1], Lingqian Liang [1], Rui Hong [1], Huiyou Chen [1], Yi Yan [1], Ling Wang [1,2], Fuyuan Zuo [1,2] and Gongwei Zhang [1,2]

Supplementary material

Table S1. Table of 27 tissue-specific metabolites in testis and liver.

**Index**	**Compounds**	**Class**	**presence in testism or liver**
MEDN098	2-Picolinic Acid	Pyridine And Pyridine Derivatives	Liver
MEDN158	Deoxyguanosine	Nucleotide metabolomics	Testis
MEDN170	Uridine 5’-Diphosphate	Nucleotide metabolomics	Liver
MEDN293	3-Hydroxypropanoic Acid	Organic Acid And Its Derivatives	Liver

MEDN324	L-3-Phenyllactic Acid	Organic Acid And Its Derivatives	Testis
MEDN338	Phenyllactate (Pla)	Organic Acid And Its Derivatives	Testis
MEDN339	Phenylpyruvic Acid	Organic Acid And Its Derivatives	Testis
MEDN378	γ -Linolenic Acid(C18:3N6)	Lipids_Fatty Acids	Liver
MEDN561	N-Acetylaspartylglutamic acid	Organic Acid And Its Derivatives	Testis
MEDN573	GDP-L-fucose	Nucleotide metabolomics	Testis
MEDN602	deoxyguanosine 5'-monophosphate (dGMP)	Nucleotide metabolomics	Liver
MEDN620	Glucotropaeolin	Carbohydrate metabolomics	Liver

MEDN704	Oxaloacetic acid	Organic Acid And Its Derivatives	Liver
MEDN724	Aspirin	Organic Acid And Its Derivatives	Testis
MEDN777	5,6-DiHETrE [(±)5,6-dihydroxy-8Z,11Z,14Z-eicosatrienoic acid]	Oxidized lipid	Liver
MEDN823	TRP-GLU	Amino Acid metabolomics	Liver
MEDN862	Phenylpyruvate	Benzene and substituted derivatives	Testis
MEDP047	Histamine	Polyamine	Liver
MEDP072	N-Isovaleroylglycine	Amino Acid metabolomics	Liver
MEDP074	N-Propionylglycine	Amino Acid metabolomics	Liver

MEDP155	5-Methylcytosine	Nucleotide metabolomics	Testis
MEDP175	Pyrimidinefreebase	Nucleotide metabolomics	Liver
MEDP295	4-Acetamidobutyric Acid	Organic Acid And Its Derivatives	Testis
MEDP545	2-Pyrrolidinone	Benzene and substituted derivatives	Testis
MEDP602	1-(4-Methoxyphenyl)-2-propanone	Benzene and substituted derivatives	Testis
MEDP879	N-Methyl-D-Aspartic Acid	Amino Acid metabolomics	Liver
MEDP889	Cortisol	Lipids	Testis

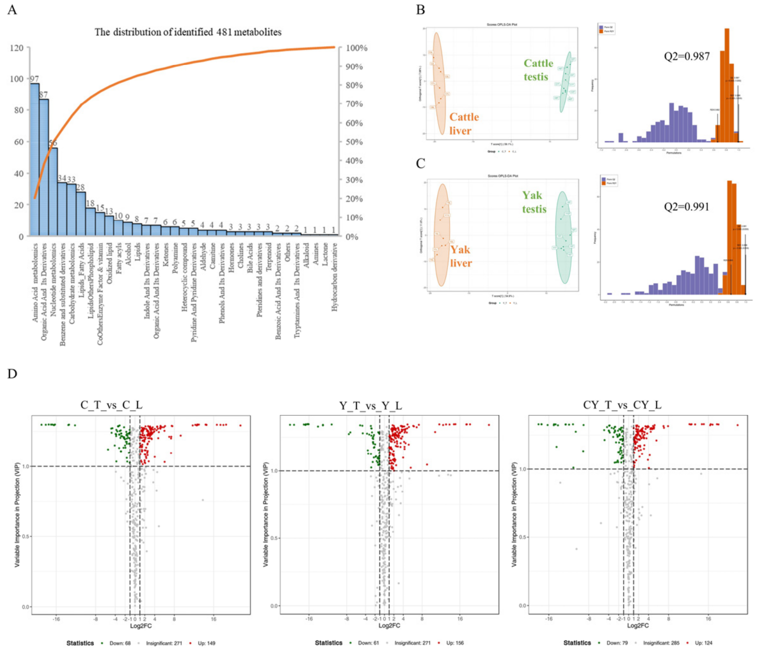

Figure S1: The distribution of identified 481 metabolites (Figure S1A); Scores OPLS-DA Plot (Figure S1B and Figure S1C); Differential Metabolite Screening (Figure S1D);
